# Intrusive Memories of Trauma in the Laboratory: Methodological Developments and Future Directions

**DOI:** 10.1007/s40473-018-0141-1

**Published:** 2018-01-31

**Authors:** Alex Lau-Zhu, Emily A. Holmes, Kate Porcheret

**Affiliations:** 1 Medical Research Council Cognition and Brain Sciences Unit, School of Clinical Medicine, University of Cambridge, Cambridge, UK; 2 Social, Genetic and Developmental Psychiatry Centre, Institute of Psychiatry, Psychology and Neuroscience, King’s College London, London, UK; 3 Division of Psychology, Department of Clinical Neuroscience, Karolinska Institutet, Stockholm, Sweden; 4 Nuffield Department of Clinical Neurosciences, University of Oxford, Oxford, UK

**Keywords:** Intrusive memories, Trauma, PTSD, Involuntary memory, Mental imagery, Trauma films

## Abstract

**Purpose of the Review:**

Intrusive memories are those that spring to mind unbidden, e.g. sensory recollections of stressful/traumatic events. We review recent methods to monitor intrusions of a stressor (a trauma film) within the laboratory.

**Recent Findings:**

Recent studies suggest three main methodologies after viewing a trauma film by which to monitor intrusions in the laboratory: during post-film rest periods, after exposure to trigger cues, and while performing an ongoing task. These approaches allow factors to be tested (e.g. psychological or pharmacological) that may influence the frequency of occurrence of intrusions.

**Summary:**

We raise methodological considerations to guide trauma film studies using intrusion monitoring in the laboratory to complement monitoring approaches in daily life (e.g. diaries). Intrusion monitoring in the laboratory also confers greater experimental control and may open novel research avenues, to advance intervention development to mitigate problematic intrusive memory symptoms.

## Introduction

Research on intrusive memories (typically of negative or traumatic events), or more simply ‘intrusions’, has expanded over the last decade, owing to an increased recognition of their role in emotional psychopathology [1–3]. In the context of treatment and prevention research for mental health, intrusions have been recently highlighted as potential intervention targets in their own right [4] and also as intermediate clinical targets which may possibly ‘knock out’ further clinical symptoms [1, 2, 5]. There is a demand for innovative approaches to reduce intrusive cognitions across psychopathology, requiring tailored methods to track and study intrusion development, persistence and mitigation. Alongside the often-used intrusion monitoring approaches in everyday life (e.g. with a diary), intrusion monitoring in the laboratory allows for additional approaches to be explored.

### The Clinical Phenomena

Intrusive memories are those that spring to mind unbidden, e.g. sensory recollections of stressful or traumatic events [6••, 7, 8]. These are common following psychological trauma [9], representing a core symptom of post-traumatic stress disorder (PTSD) and acute stress disorder (ASD) [10]. For instance, a trauma survivor after a gun assault may repeatedly experience a vivid mental image of ‘a gun put to the head’ [8]. Intrusive manifestations are distinct from voluntary retrieval [11•, 12, 13], for example, when the same trauma survivor deliberately recalls details of the attack to participate in a court case and describes what happened during the trauma (although they may also experience intrusions once they have started deliberately recounting the trauma).

### Experimental Psychopathology: the Trauma Film Paradigm

Experimental psychopathology [14], or more generally experimental medicine, can be used as an approach for innovation in the prevention and treatment of mental health difficulties, particularly at preclinical stages of intervention development. The approach aims to model clinical processes under controlled laboratory conditions. For a better understanding of the impact of psychological trauma (e.g. for potential ASD or PTSD), a experimental psychopathology model would be of benefit if it could simulate both exposure to trauma and generate a form of its hallmark symptom—intrusive memories of the traumatic event.

**Fig. 1:**
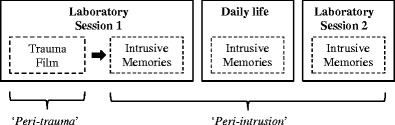
Basic procedure of a typical study using the trauma film paradigm. Trauma film (i.e. film with traumatic content) is presented in the laboratory (session 1); intrusive memories are typically monitored in daily life (e.g. over several days via diary). Recent studies, as shown in the current review, have also included methods to monitor intrusive memories in the laboratory (over several minutes). Intrusive memory monitoring in the laboratory can take place within the same session as the film and/or at a later session. ‘Peri-trauma’ means during viewing of the film, and ‘peri-intrusion’ means during monitoring of intrusive memories

The ‘trauma film paradigm’ emerged as an experimental model of intrusions generated in response to a laboratory stressor in the 1960s, initially pioneered by Horowitz [15] and Lazarus [16]. The paradigm involves participants watching film footage depicting stressful/ potentially traumatic events (i.e. modelling exposure to trauma), which are powerful enough to induce intrusions of the film in everyday life for up to several days outside the laboratory (i.e. modelling intrusive symptoms) [17••] (Fig. 1). Interestingly, this paradigm could in the future also facilitate translational links with other human and non-human models such as fear conditioning paradigms, as similar aspects of emotional responding can be assessed across paradigms, e.g. psychophysiological outcomes [11•].

The trauma film paradigm provides researchers with a platform to test proof-of-concept innovative interventions to, for example reduce or increase the frequency of intrusions, evaluate risk factors for intrusion development and explore mechanisms by which interventions could worsen stress symptoms [18–26]. Findings from the laboratory using this paradigm have shown preliminary evidence of translation to real-life settings. For instance, a behavioural protocol (a memory reminder cue followed by the computer game Tetris) was used in the laboratory soon after exposure to a trauma film with non-clinical volunteers. Such a protocol was hypothesised to interfere with the (re)consolidation of memories (by competition with cognitive resources), which would otherwise become intrusive [18, 27, 28]. Compared to a control condition, this intervention protocol was found to reduce intrusions as monitored in a 1-week diary [28]. A similar protocol used with patients following a real-life road traffic collision [29••] and with mothers after an emergency caesarean section [30] also led to fewer intrusions for the 1-week period post-trauma compared to control protocols.

#### Monitoring Intrusive Memories in Daily Life

The most common approach for assessing intrusions to a trauma film is to sample intrusions after participants have left the laboratory and gone back to everyday life (Fig. 1). Typically, participants return to their daily lives after viewing the film and record the intrusions they experience using pen and paper [18, 31] or electronic daily diaries [32, 33] for some days. A benefit of real-life monitoring is that it allows us to study intrusions in an everyday context that is potentially more akin to the context in which intrusions of real-life traumas occur. It also allows us to examine if an intervention delivered within the laboratory has a subsequent impact on cognitive experiences outside of the laboratory. However, sampling intrusions in everyday contexts does come with some drawbacks, including possible non-compliance with completion of monitoring methods and variability in the contexts experienced by individuals in their everyday lives (e.g. environmental cues and/or daily activities).

#### Monitoring Intrusive Memories in the Laboratory

A complementary approach to real-world diaries is monitoring involuntary cognitions in the laboratory [34, 35]. Lab-based assessments of intrusive memories have been used since the early trauma film studies in the 1970s [15] as well as over the last decade [17••] (Fig. 1). Unlike diaries, intrusion monitoring in the laboratory can provide additional experimental control over the retrieval context for trauma film studies. For example, contextual factors such as environmental cues and/or the attentional state of the participant while intrusions arise can be controlled for, potentially reducing inter-individual variability. Moreover, these contextual factors can also be directly manipulated, so their impact on intrusion retrieval (and how such context might interact with the effect of the primary factor of interest) can be tested. Currently, a review of methods to monitor intrusions (of an experimental trauma) within the laboratory is lacking.

## Aim and Scope

We aim to summarise recent methodological developments using the trauma film paradigm that allow for monitoring of intrusive memories in the laboratory. To this end, we selectively reviewed the relevant literature in the last decade [17••], focussing on studies that (a) induced intrusive memories using stressful films, (b) studied the frequency of occurrence of intrusions and (c) assessed intrusions both within a laboratory setting and in daily life to allow comparison. We hope to illustrate the numerous possibilities for monitoring intrusions of trauma films within the laboratory. Later in the *Conclusions*, we raise methodological considerations for incorporating intrusion monitoring in the laboratory within the trauma film paradigm, highlight future research directions using such methodologies and discuss considerations for conducting research using the paradigm more broadly.

## Methodological Variations in Monitoring Intrusive Memories in the Laboratory

Mirroring the use of the clinical term *peri-trauma* to refer to the period during the traumatic event, we will use the term *peri-intrusion* to refer to the period during which intrusions are monitored and assessed (Fig. 1). Three key parameters were identified that have been used to simulate the peri-intrusion window in the laboratory: (a) whether a definition of intrusions was provided to participants before or after the peri-intrusion window, (b) whether or not triggering cues were presented and (c) whether or not an ongoing task was included. Different combinations of these parameters yielded three main intrusion-monitoring methodologies in the laboratory (Table 1): (1) intrusions that occur during post-film rest periods (three studies), (2) those occurring in the context of triggering cues (ten studies) and (3) those that occur while participants are performing an ongoing task (four studies).

**Table 1:** A selective review of studies where both viewing of a trauma film and monitoring of intrusive memories took place within the laboratory

Study	*N*	Design	IV of primary interest	Timing of IV	Intrusive memory sampling in the laboratory (DV: Frequency of occurrence of intrusions)				
					Peri-intrusion duration	Estimation method by the participant	Trigger cues	Ongoing task	Timing of the peri-intrusion period within the experimental design
Type 1: rest periods									
Wilksch and Nixon [36]	49	Correlational	Prior negative cognitions	–	5 min	Real time	–	–	Day 1: soon after film and Day 8
Hawkin and Couglas [37]	54	Experimental	Acute nicotine administration	Before film	5 min	Real time	–	–	Day 1: soon after film
Clark et al. [38]	35	Experimental	Button presses associated with intrusions	After film	6 min	Real time	–	–	Day 1: soon after film
Type 2: trigger cues									
Schaich et al. [41]	66	Experimental	Abstract/concrete processing	Before film	5 min	Retrospective	Visual	–	Day 1: soon after IV
Ehring et al. [42]	101	Experimental	Abstract/concrete processing and distraction	After film	5 min	Retrospective	Visual and auditory	–	Day 1: soon after film
Morina et al. [31]	67	Correlational	Trait general use of imagery	–	2 min	Retrospective	–	–	Day 1: soon after film
Wegerer et al. [43]	66	Experimental	Conditioned and unconditioned cues	After film	3 min	Retrospective	Auditory	–	Day 1: 30 min after film
Marks and Zoellner [44]	148	Experimental	Extinction procedures	After film	25 s	Retrospective	Auditory	–	Day 3
Malik et al. [45]	110	Correlational	Hypomanic experiences	–	2 min	Real time	Visual	–	Day 8
Lang et al. [46]	48	Experimental	Cognitive bias modification procedure	Before film	2 min	Real time	Visual	–	Day 8
James et al. [18], Exp 1	52	Experimental	Reminder plus Tetris game play procedure	After film	2 min	Real time	Visual	–	Day 8
James et al. [18], Exp 2	76	Experimental	Reminder plus Tetris game play procedure	After film	2 min	Real time	Visual	–	Day 8
James et al. [47]	56	Experimental	Tetris game play procedure	Before film	2 min	Real time	Visual	–	Day 8
Type 3: ongoing tasks									
Verwoerd et al. [48]	45	Experimental	Attentional training	After film	3 min	Real time	–	Focus on breathing	Day 1: soon after IV
Marks et al. [44]	49	Correlational	Analogous psychotic experiences	–	4 min	Real time	–	Digit monitoring	Day 1: soon after film
Nixon et al. [50]	120	Experimental	Thought suppression and cognitive load	After film	5 min	Real time	–	Suppression and/or cognitive load	Day 1: soon after film and Day 8
Nixon et al. [51]	80	Experimental	Thought suppression and cognitive load	After film	5 min	Real time	–	Suppression and/or cognitive load	Day 1: soon after film and Day 8

Studies are presented in the order as they appeared in the main text. Peri-intrusion means during intrusion monitoring

*N* number of participants in the main analyses, *IV* independent variable, *DV* dependent variable

### Intrusive Memories During a Post-film Rest Period

One method for sampling intrusions in the laboratory is during periods of quite rest, typically with eyes closed for roughly 2 to 5 min. A definition of an intrusive memory is given to participants prior to the post-film rest period and they are instructed to specifically monitor intrusions as they happen in real-time (Table 1). Using this approach, Wilksch and Nixon [36] assessed intrusions during a 5-min peri-intrusion period, first immediately after a trauma film and then 1 week later in a separate laboratory session. For each peri-intrusion period, participants were instructed to lift a finger when an intrusion occurred and lower their finger when the intrusion had gone. Finger movements during these periods were videotaped and later analysed. Individuals with a tendency to interpret intrusive symptoms more negatively, compared to those who did not, reported subsequently more laboratory intrusions (both rest periods) and in their everyday intrusions (1-week diary completed between both sessions).

A 5-min peri-intrusion period was also employed by Hawkins and Cougle [37]. Soon after the film, participants completed a free recall task and a recognition memory task regarding the content of the trauma film and then used a tally counter to monitor intrusions during the peri-intrusion period. Individuals who underwent acute nicotine administration prior to film viewing, compared to a placebo lozenge group, reported more laboratory intrusions within the first session but not in a subsequent 1-week diary.

Clark et al. [38•] assessed the neural correlates of intrusive memory *retrieval* using functional magnetic resonance imaging (fMRI). Immediately after watching a film in the fMRI scanner, participants were asked to press a button if they experienced an intrusion of any scene from the film while remaining in the scanner for 6 min (peri-intrusion period). To minimise experimental demands, they were told not to worry if they did not experience any intrusions. Brain activation related to intrusion key presses was compared to brain activation associated with random key presses generated by a separate group of participants who did not watch the film. Experiencing an intrusion was associated with brain activity in frontal regions, but most notably in the left inferior frontal gyrus, an area also implicated in the initial *encoding* of specific film scenes that subsequently intruded, as indicated by intrusion descriptions in a 1-week diary.

### Intrusive Memories After Exposure to Trigger Cues

Although intrusions usually appear to spring to mind unbidden, clinical theories propose that these are often triggered by reminders that have sensory-perceptual overlap with the initial encoded event [39, 40]. Drawing from these perspectives, a number of trauma film studies have sampled intrusions while exposing participants to reminder cues from the trauma film (Table 1). For example, Schaich et al. [41] asked participants to undergo two 3-min rest periods after film viewing: a first one without any cues (uncued rest) and a second one after exposure to nine visual stills (presented for 10 s each) taken directly from the film (cued rest). Participants estimated the total number of intrusions experienced at the end of each rest period and also every evening for the subsequent 7 days in daily life. Intrusion count in the laboratory was reported by collapsing both rest periods. Higher *trait* rumination was found to be associated with more frequent intrusions both in the laboratory and daily life for individuals who were trained to use abstract processing (focussing on meanings) but not concrete processing (focussing on the events) *before* film viewing.

A similar approach using retrospective assessment was adopted by two additional studies, with findings reported instead separately for uncued and cued rest periods. For cued rest, Ehring et al. [42] used auditory cues and visual stills from scenes of the original source of the film footage which did not overlap with the scenes shown to the participants. Participants were assigned to one of three guided thinking tasks immediately after the film, abstract, concrete or distraction and then underwent the uncued rest followed by the cued rest. For cued rest, the concrete thinking group reported fewer intrusions than the distraction group, with the abstract group lying numerically in the middle. However, no significant group differences in intrusion frequency were reported for uncued rest or in daily life (3 days post-film), suggesting that concrete thinking may modulate the ability of cues (at least in the laboratory) to trigger intrusions. In contrast, Morina et al. [31] reported a similar pattern of results for intrusions in both uncued and cued rests. Participants underwent 2-min rest periods twice, once immediately after the film (uncued), and followed by another after exposure to seven still pictures from the film (cued). Higher trait mental imagery vividness was associated with more frequent laboratory intrusions (in both rest periods) and also more intrusions in a 5-day diary, suggesting that the level of trait imagery vividness is a potential risk marker for increased intrusions after a stressor.

Using a Memory Trigger Task, Wegerer et al. [43•] developed an innovative approach to trigger intrusions within the same session as film viewing. After watching a trauma film, participants listened to three types of sound landscapes: (1) embedded with an auditory cue associated with the trauma film (conditioned cue), (2) embedded with an auditory cue not associated with the film (unconditioned cue) or (3) not embedded with auditory cues (no-cue control). After each landscape, participants retrospectively estimated the total number of intrusions. The conditioned cue elicited more intrusions in the laboratory, as well as higher skin conductance levels and anxiety ratings, compared to the unconditioned cue or no-cue control. A higher negative rating to the conditioned cue was also associated with more intrusions in daily life (estimated in each of the subsequent three evenings). This study illustrates the advantage of sampling intrusions in the laboratory to investigate concurrent correlates of emotional responding, including psychophysiological outcomes.

Marks and Zoellner [44•] also developed a novel method to assess intrusive memories. Participants initially watched a trauma film in the laboratory and 2 days later returned for an extinction manipulation. At 24 h after the manipulation, participants received a phone interview: they first estimated the overall number of intrusions experienced over the last 24 h pre-interview; they were then presented with a Fear Renewal Task, during which they closed their eyes and paid attention to a 25-s audio clip directly obtained from the film; at the end of the clip, participants estimated the number intrusions experienced both during and after the audio clip. The number of intrusions post-manipulation was collapsed across all the above monitoring stages. It was found that an extinction intervention led to more intrusions than did control procedures. This study showcased a creative solution to provoke intrusions under experimental control (stimuli presentation via the telephone) while minimising the potential burden of returning to the laboratory.

All the above studies relied on retrospective estimates by participants. To assess intrusions throughout the peri-intrusion window, various studies have employed the Intrusion Provocation Task (IPT) [45, 46]. Here, participants are first presented with film-related visual cues, which consist of film stills of neutral scenes from the film (e.g. stills that do not depict the ‘worst’ moments, e.g. the car collision). They are then instructed that they can think freely without restrictions for 2 min during a rest period. Participants then indicate each intrusion occurrence as they happen, via keyboard button presses or tally markers on paper. All of the following studies used the IPT 1 week after the film in a second laboratory session. Malik et al. [45] found that young people with a high incidence of hypomanic experiences, compared to controls, reported more intrusions in the IPT as well as in a 1-week daily sampling of intrusions via text message. Lang et al. [46] found that a positive appraisal training after a film led to fewer intrusions reported in the IPT and in a 1-week diary compared to a negative appraisal training. In two experiments, James et al. [18] found that a behavioural protocol (film reminder cue before a 10-min gap followed by Tetris game play at 24 h post-film) led to fewer intrusions both in the IPT and in the 1-week diary compared to control protocols (reminder-only, Tetris-only or no-task controls). A subsequent study by James et al. [47] found that a similar behavioural protocol administered *before* film viewing did not influence intrusions (in either the IPT or a 1-week diary), suggesting temporal constraints of this type of interventions such that it may be effective if delivered after but not before trauma exposure.

### Intrusive Memories While Performing an Ongoing Task

The third approach to assess intrusions in the laboratory is during ongoing tasks as opposed to pure rest periods, potentially creating a situation more akin to when intrusions occur alongside other activities in everyday life. Typically, participants are instructed to notice intrusions while performing the ongoing task, indicating each intrusion occurrence in real-time using keyboard button presses or a tally counter (Table 1). For example, Verwoerd et al. [48] assessed laboratory intrusions while participants were also instructed to actively focus on their breathing during a 3-min period. Participants who were trained to direct their attention away from film reminders after viewing the film, relative to those who received a control training, reported fewer laboratory intrusions after the training within the same session, as well as in a subsequent 3-day diary.

Marks et al. [49] assessed laboratory intrusions while participants performed a concurrent 4-min digit task. This task involved a random series of two-digit numbers being presented on a computer screen, which participants were instructed to read out loud. Simultaneously, participants indicated each intrusion occurrence with a handheld clicker. The study explored the effect of a visuospatial task (versus no task) during film viewing on intrusions but found no effect of condition on either laboratory intrusions (30 min post-film) or daily life intrusions in a 1-week diary. However, it was found that participants who reported having psychotic-like experiences in daily life, compared to those who did not, reported more laboratory and diary intrusions, suggesting that psychotic-like experiences may confer vulnerability to intrusion development.

Two additional studies manipulated the type of ongoing task to directly examine their impact on the number of intrusions concurrently experienced as well as those experienced later. For the peri-intrusion periods, participants were asked to close their eyes and then lift a finger when an intrusion occurred and lower the finger when the intrusion was gone (similar to the approach used in studies with pure resting periods). These periods were videotaped and scored later. Intrusions were monitored in the first session and in a later session at 1 week after the film, but this second time without performing the ongoing tasks. A 1-week intrusion diary was completed in daily life between sessions. Using such an approach, Nixon et al. [50] assessed intrusions while participants performed one of the following ongoing tasks soon after the film: suppressing any film-related thoughts, suppression while also holding one of three cognitive loads (hyperventilation, visuospatial load or verbal load) or no suppression at all. Findings showed that there were no significant group differences on the number of laboratory or diary intrusions. In a second study [51], participants underwent the intrusion assessment after viewing the film (and completing a word-stem task and dot-probe task with film-related information) while simultaneously performing similar tasks to the first study: suppressing any film-related thoughts, holding a verbal cognitive load, both suppression and holding a cognitive load or neither. Again, findings showed no significant group differences on intrusion frequency immediately after the film or at 1 week. However, individuals who performed both suppression and holding a cognitive load during the first 5-min period subsequently reported instead more diary intrusions.

## Conclusions

A review of the literature of recent studies using the trauma film paradigm has revealed three main methodologies to monitor intrusive memories within the laboratory: (1) during post-film rest periods, (2) after exposure to triggering cues or (3) while performing ongoing tasks. A primary focus of this research to date is testing associations between relevant factors (e.g. psychological or pharmacological) before, during or soon after trauma and the frequency of intrusions at a later time point. Next, we discuss key methodological considerations to guide experimental design using intrusion monitoring in the laboratory, complementing monitoring approaches in daily life (e.g. diaries). Then, we argue that intrusion monitoring in the laboratory is yet to be fully exploited and can be leveraged for novel avenues in experimental psychopathology research, including research into the context in which intrusions arise and the impact of intrusions themselves. Finally, we discuss limitations of the trauma film paradigm and issues associated with its implementation.

### Methodological Considerations

One consideration is when the peri-intrusion window (i.e. the time when intrusions are monitored) occurs in relation to other tasks within the full experimental design. In some studies, the intrusion-monitoring period may be preceded by one or more tasks related to another aim of the study. For instance, intrusion-monitoring could be preceded by a word-stem task and/or a dot-probe task with verbal information related to the film [51]. These tasks may provide reminders about the film and that could then potentially act as triggers for intrusions. It is important that these unintended triggers do not inadvertently lead to intrusion ‘over-provocation’, i.e. a ceiling effect that could mask the association with the primary factor of interest. Additionally, tasks that elicit voluntary memory (e.g. free recall and/or recognition) may activate a ‘voluntary’ retrieval mode, potentially making it more difficult to ascertain if the intrusions subsequently reported were indeed ‘involuntary’ [37]. Critically, voluntary retrieval of film content itself can also modulate intrusions [52, 53]. Thus, it is preferable to administer tasks with information about the film *after* the peri-intrusion window whenever possible or consider counterbalancing the order of such tasks with intrusion monitoring. Alternatively, a more rigorous but also laborious approach (due to the increased sample size needed) is to use a between-subject design where each group is administered one type of memory test only. Furthermore, researchers may also be cautious about eliciting verbal descriptions after the occurrence of each intrusion within the laboratory—while this may provide richer descriptions of the intrusion content, it may also act as a form of additional voluntary retrieval that can inadvertently influence the subsequent rate of intrusions [52, 53].

Another consideration is the use of rest periods versus ongoing tasks during the peri-intrusion window. Rest periods are easy to implement. However, ongoing tasks could increase experimental control over the retrieval phase, e.g. equating attentional instructions across participants [48, 49]. If such tasks are considered, then it is important that these are not extremely taxing as the development of intrusions could be impeded all together, leading to a floor effect. Studies reviewed here typically used tasks with low attentional demands such as breathing [48] and digit monitoring [49].

Real-time monitoring of intrusions is generally preferred, as retrospective estimates may suffer from memory biases. With a clear a priori definition of what constitutes intrusive memories, monitoring can become relatively simple for the participant, who can then easily distinguish intrusions from other related processes (e.g. intrusive versus voluntary memories) [18, 45–47]. However, retrospection may be preferable in some designs where uninterrupted performance on a primary task is needed (e.g. task with reaction time measures). In such cases, retrospective biases should be minimised with appropriate durations of monitoring, e.g. most studies in this review used no more than 3 to 5 min of monitoring before participants reported retrospective estimates [31, 41–43].

It is also important to consider the timing of intrusion monitoring in the laboratory, which could take place within the first session (e.g. immediately after, or after a short period) or in a subsequent session (Fig. 1). Such timing should be informed by mechanistic theory of how the primary variable of interest relates to emotional memory over time. For instance, if the impact of an intervention on memory takes time to emerge, e.g. due to consolidation [54] or reconsolidation processes [55], effects on intrusion monitoring immediately after the intervention would not be expected, so later monitoring periods would also be needed to track such potential time-dependent effects.

Finally, we must carefully evaluate the distinction between ‘uncued’ versus ‘cued’ intrusions. While clinical proposals suggest a primary role of sensory-perceptual cues in the development of intrusive symptoms [32, 33], the assumption that such cues are necessary to sample intrusions in the laboratory has been little researched. Interestingly, one may argue that the occurrence of intrusions during ‘uncued’ rest periods indicate that overt cues are not always necessary to provoke intrusions. However, triggers may also arise from testing participants in the same context as film viewing (e.g. same room, researcher and/or apparatus including brain-imaging apparatus), other tasks (containing trauma-related information) within the experimental design as described previously and/or internal cues (e.g. mood or arouse states). It remains to be established whether or not (and which) triggers are important to sample intrusions, and whether those triggered specifically by sensory-perceptual cues are the most relevant to the clinical phenomena. Thus, it may be important to assess intrusions and their associated triggers whenever possible, as these may inform the potential mechanisms of putative intervention.

### Future Directions

The main value of using laboratory monitoring compared to diaries in trauma film studies is the additional control over *retrieval* processes that pertain to intrusions. This opens up numerous new research directions. First, we can design experiments that elucidate the role of retrieval/contextual factors on the development and persistence of intrusions as specified by clinical and theoretical models of intrusions [39, 40, 56, 57]. These include the role of different trigger cues [43•], as described above, and the role of ongoing activities [50, 51]. Building on the studies reviewed here, a greater understanding of such retrieval processes could inform more precise parameters for designing intrusion-monitoring methods.

A second use of laboratory monitoring is to assess the causal impact of intrusions on other processes. Emerging research suggests intrusions impacts on daily functioning [4], yet most experimental psychopathology research focuses on the impact of other variables on intrusions [17••] rather than the impact of intrusions themselves. Experimentally induced intrusions in the laboratory could be used to assess their impact on other cognitive processes, e.g. concentration [58]. Concurrent physiological correlates of intrusion retrieval can also be assessed dynamically in real-time throughout the peri-intrusion window in the lab, including peripheral physiology, e.g. skin conductance and heart rate [43•], and neurophysiology, e.g. fMRI [38] and electroencephalography [59, 60].

A third use of laboratory monitoring is to make better comparisons with tests of voluntary memory (e.g. free recall or recognition memory). It is desirable to reduce intrusions without interfering with voluntary memory of an event, e.g. in order to be able to provide clear legal accounts of a traumatic event [11•], and thus, it is of interest to assess the impact of interventions on both memory types. Tests of voluntary memory are typically performed in the laboratory, whereas intrusions are mostly assessed outside of the laboratory [17••]. Having laboratory methods to monitor intrusions means that both memory types can be assessed in tandem while matching potential confounds, e.g. similar amount of triggering cues and attentional focus across test types.

One observation from this review is that in some studies [37, 41, 50], a primary variable of interest shows significant associations with the number of laboratory intrusions only or instead with only the number of daily intrusions. One reason may be due to the limited statistical power. Another reason may be due to methodological differences in, for example retrieval delays (laboratory intrusions typically cover early time periods whereas diary intrusions cover later periods) [37, 38•] or availability of coping strategies (one may be more likely to engage in suppression in the laboratory but distraction in daily life) [36]. More research is needed to understand differences and similarities between both sampling contexts. Such research would also benefit from better establishing the psychometric properties of both monitoring methodologies.

Finally, monitoring intrusions in the laboratory also offers practical advantages for future experiments. A trauma film study typically requires two sessions separated by usually a 1-week diary. Instead, a study design that can consider trauma film and intrusion monitoring within a single session could reduce potential participant burden, avoid dropouts and speed up data collection. Such single-session experiment also opens up the possibility of inducing and then dampening intrusions by the end of the session, which may facilitate research with clinical populations where the importance of intrusive imagery is becoming increasingly recognised [61, 62].

### Limitations of the Trauma Film Paradigm

There are several limitations of the trauma film paradigm. Even though it may have greater ecological validity than, for example fear conditioning to abstract stimuli or viewing still pictures, viewing films with traumatic content in the laboratory is clearly not the same as experiencing real-life trauma or witnessing trauma indirectly in the line of work. In recent years, there has been increased recognition of the role of indirect media exposure of traumatic events on psychopathology in civilians [63, 64]. Further in a professional context, DSM 5 criteria [10] appear to suggest that viewing such footage could comprise indirect exposure to trauma within a diagnosis for PTSD, e.g. a police officer reviewing video footages of child trafficking. While the trauma film paradigm provides a model to study the impact of viewing trauma more broadly, it does not aim to simulate, for example media-based exposure that is repeated and prolonged or trauma footage in its most extreme real-life form. Therefore, experimental psychopathology findings from the lab must be complemented with prospective studies of exposure to real-life trauma (e.g. [63], [65]), such as in individuals who are at high risk of direct exposures (e.g. paramedics, fire fighters, military personnel) or indirect exposure in the line of work (e.g. police, news editors).

Measuring characteristics of intrusions to trauma films rely on self-report accounts, which, as in all self-reports, are susceptible to demand characteristics, i.e. that an experimental effect is driven by participants knowing the purpose of the study. This issue also applies to studies with patients with ASD/PTSD, who are asked to self-report their intrusive symptoms during clinical interviews, assessments and/or throughout interventions. Trauma film studies can attempt to mitigate such an issue by, for example, asking about expectations, to at least check that there are no differences in expectation between experimental conditions—that way, experimental effects can be more confidently attributed to the main variable being manipulated rather than mere demand. One motivation for including methods to monitor intrusions in the laboratory is to facilitate the development of sensitive behavioural/physiological markers of intrusive symptoms to complement self-report measures and which are potentially less susceptible to demand.

### Considerations for Conducting Research Using the Trauma Film Paradigm

It would clearly be unethical to deliberately have individuals go through actual psychological trauma. One goal in selecting film material for the trauma film paradigm might be not to present individuals with the most *distressing* film available but rather the mildest film needed to generate some intrusions, i.e. to be sufficient but not the most extreme [17••]. As a variety of films can be used [66•], some research groups may prefer not to use films of particular trauma themes, e.g. rape. Deciding what materials will and will not be used will depend on the research aims (e.g. if the aim is to study intrusion modulation rather than the psychological impact of sexual traumas). Furthermore, the ability of the film to generate intrusions may depend on additional aspects of the experimental protocol or set-up, for example specific instructions on how to view the film, apparatus used to view the film, how the researcher engages with the participant throughout testing, instructions on how to identify intrusions and distinguish them from other non-intrusive mental experiences, instructions on how to complete and return the diary to optimise compliance and accuracy, etc.

Researchers should be appropriately trained and supervised throughout the running of a trauma film study. Good practice is to seek training and materials from other researchers who have used the paradigm previously. Merely trying to use the paradigm de novo without sufficient training means that, for example it would be hard to interpret whether or not null results were due to deficits in methodological competence. A researcher or research group using this paradigm for the first time may consider a significant training period before embarking on data collection for a new study. One might also seek to test replication of pattern of results from previous research for example, rather than trying their hand to an entirely novel question with a new paradigm. However, clearly, further work needs to be done to test the best way to train and disseminate use of this experimental paradigm, and ideas are to be welcomed.

The films used are typically intended to induce only mild distress and induce some intrusions which typically subside after a few days. Nevertheless, researchers should be sensitive to distress and consider strategies to help mitigate any disproportionate distress reported by the participants [17••]. For example advertising materials and information sheets for the study should report the nature of the film, that intrusions may arise and also provide readily available contact details for the study (e.g. on the intrusion diary). Participants should be reminded of the right to terminate film viewing and/or to withdraw from the study at any point without explanation. Measures could be included to monitor their mood and anxiety levels throughout the study. One may also consider implementing safeguarding measures prior to the study, such as including a clinically qualified member of staff available to the researchers (without clinical background) for guidance throughout, and/or excluding individuals who have a history of severe mental health difficulties or trauma exposure, and so forth. Such decisions should be made on a study-by-study basis depending on the design and aim of the research.

### Final Remarks

Methodologies to monitor intrusive memories within the laboratory in studies using the trauma film paradigm have gained traction in the last few years: bringing intrusions ‘out of the wild’ and ‘into the lab’. We have identified three such methodologies to monitor intrusive memories (of trauma films) within the laboratory: during post-film rest periods, after exposure to trigger cues and while performing an ongoing task. These methodological developments may open up novel research possibilities to advance research on intervention development for psychopathology in which intrusive memories are problematic.
